# Serum α-Carotene, but Not Other Antioxidants, Is Positively Associated with Muscle Strength in Older Adults: NHANES 2001–2002

**DOI:** 10.3390/antiox11122386

**Published:** 2022-12-01

**Authors:** Renata R. Bruno, Fernanda C. Rosa, Paula C. Nahas, Flávia M. S. de Branco, Erick P. de Oliveira

**Affiliations:** Laboratory of Nutrition, Exercise and Health (LaNES), School of Medicine, Federal University of Uberlandia (UFU), Av. Para, 1.720 Bloco 2U-Sala 20, Campus Umuarama, Uberlandia 38400902, MG, Brazil

**Keywords:** antioxidants, muscle strength, aging

## Abstract

Aging is associated with an increased reactive oxygen species that can decrease muscle strength. Thus, antioxidant substances could be positively associated with muscle strength in older adults. To investigate the association between serum antioxidants and muscle strength in older adults. A cross-sectional study evaluating 1172 individuals (627 men and 545 women), aged 50 to 85 years from NHANES 2001–2002, was performed. Carotenoids (α-carotene, trans-β-carotene, cis-β-carotene, β-cryptoxanthin, lutein/zeaxanthin combination, trans-lycopene), vitamin E, and retinol were analyzed via the high-performance liquid chromatography method. Muscle strength was evaluated by the isokinetic knee extension test. Linear regression was performed to evaluate the association between tertiles of serum antioxidant levels and strength, adjusted for confounders (energy and protein intake, body mass index, sex, age, C-reactive protein, uric acid, race/ethnicity, marital status, annual household income, educational level, physical activity, smoking, hypertension, arthritis, and diabetes). Alpha-carotene levels (*p*-trend = 0.027) were positively associated with muscle strength. However, serum vitamin E, trans-β-carotene, cis-β-carotene, β-cryptoxanthin, carotenoids, and retinol levels were not associated with strength. Serum α-carotene, but not other antioxidants, was positively associated with muscle strength in older adults.

## 1. Introduction

Aging is associated with muscle strength loss [1], which increases the risk of mortality [2,3], is associated with an increased risk of falls [4] and fractures [5,6], as well as a lower ability to perform activities of daily living [7]. The decrease in strength is influenced by several factors, such as changes in eating patterns [8], anabolic resistance [9], comorbidities [10], reduced physical exercise practice, and increased oxidative stress [11,12,13].

Since increased oxidative stress seems to be one of the causes of muscle strength loss in older adults, substances with antioxidant properties could decrease the oxidative stress [14,15], maintaining the strength over time [16]. Carotenoids, vitamin E, and retinol are potent antioxidants that can decrease the oxidative stress, and thus can be positively associated with muscle strength [16,17,18,19,20,21,22]. Semba et al. [23] evaluated the association of plasma carotenoids and α-tocopherol with grip, hip and knee strength in older women. Higher levels of carotenoids and α-tocopherol were associated with higher muscle strength [23]. In a prospective cohort study, Sahni et al. [16] showed that the intake of total carotenoids, lycopene, and lutein + zeaxanthin was positively associated with handgrip strength. However, to date, the evidence is still limited regarding the association of serum antioxidants with muscle strength, as only a few studies have evaluated this topic [16,23]. In addition, the methodology of the studies is heterogeneous, since one study evaluated only women [23] and another study evaluated the consumption of antioxidants by conducting a food frequency questionnaire, and not by observing the concentrations of these antioxidants in the plasma [16]. Therefore, more studies evaluating the association between serum antioxidants and strength in older adults are needed. The aim of the present study was to assess the association between serum antioxidants and muscle strength in older adults from the National Health and Nutrition Examination Survey (NHANES) 2001–2002. We hypothesized that higher levels of serum carotenoids, vitamin E, and retinol would be associated with higher muscle strength.

## 2. Methods

### 2.1. Participants

**Figure 1:**
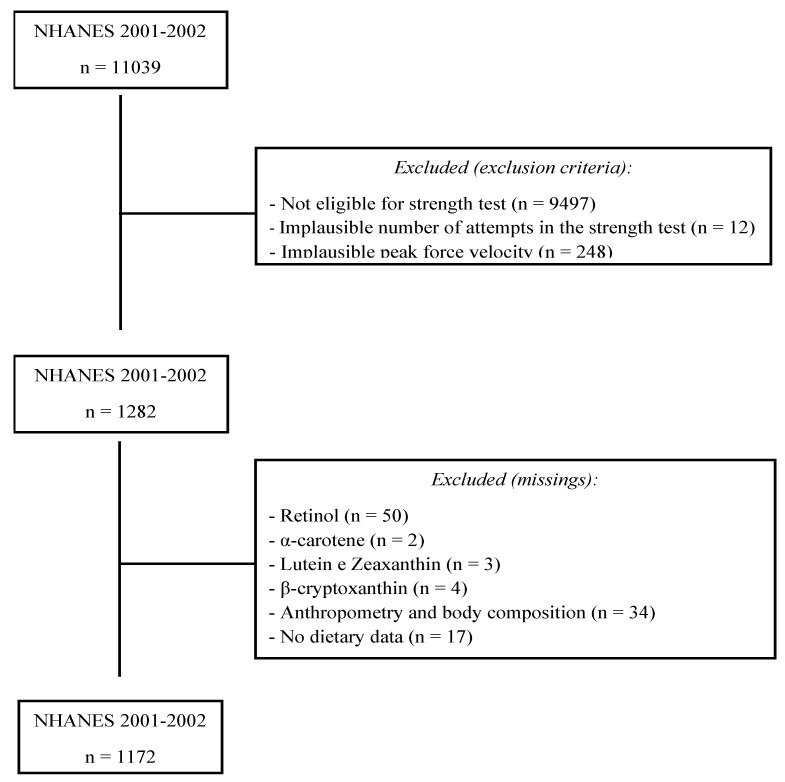
Flowchart of the sample selection from NHANES 2001–2002.

This is a cross-sectional study conducted with NHANES data from 2001–2002. The NHANES is a survey developed by the National Center for Health Statistics (NCHS), comprising several assessments of health and the nutritional status of a representative sample of the non-institutionalized population of the United States. In NHANES 2001–2002, 11,039 individuals were evaluated; however, in the present study, individuals with missing data in the isokinetic strength test, serum vitamin A and carotenoids, dietary intake, and anthropometry, were excluded. In addition, participants with implausible peak force velocity (<55°/s or >65°/s) [24] and who did not perform at least 4 trials in the isokinetic strength test, were excluded from the study. Therefore, 1172 individuals (627 men and 545 women), aged between 50 and 85 years, were included in the present study (Figure 1). The age range was chosen due to the eligibility of the strength test and because it is an age range in which it is already possible to observe an important muscle strength loss [25]. Written consent, as well as approval from the Research Ethics Review Board of the NCHS (protocol n° 98-12 for the NHANES 2001 and 2002 cycle), were obtained from all NHANES participants.

### 2.2. Muscle Strength

The peak isokinetic knee extensor strength was evaluated using six measurements from the right quadriceps, performed at a speed of 60° per second and using the Kinetic Communicator isokinetic dynamometer (Kin Com MP, Chattecx Corp., Chattanooga, TN, USA). The first three trials were used for learning the movement and warming up; therefore, the participants were instructed not to apply maximum strength. In the last three trials, they were encouraged to perform with maximum effort for the muscle strength assessment. Participants who had extreme peak-force velocity values (<55°/s or >65°/s) [24] were excluded, and for individuals who completed 4–6 trials, the highest value was used.

### 2.3. Serum Antioxidants

Vitamin E, retinol, α-carotene, trans-β-carotene, cis-β-carotene, β-cryptoxanthin, lutein/zeaxanthin combination, and trans-lycopene were the antioxidants evaluated in the blood. The sum of α-carotene, trans-β-carotene, cis-β-carotene, β-cryptoxanthin, lutein/zeaxanthin combination, and trans-lycopene were considered for total carotenoids. The high-performance liquid chromatography method, with photodiode array detection, was used for the assessment of serum antioxidants. In order to obtain more reliable results, a fasting sample was obtained and the exposure of the serum to sunlight or other sources of full spectrum radiation was avoided [26]. Serum antioxidant data were expressed in µmol/L, with the exception of retinol, which was expressed in µg/dL.

### 2.4. Anthropometry

The Lohman protocol [27] was used for the analysis of body weight and height, and the body mass index (BMI) was calculated by body weight divided by height squared.

### 2.5. Dietary Intake

For the dietary intake assessment, a 24-h food recall was performed. In NHANES 2001, dietary intake was evaluated through the 4-step multiple pass (quick unstructured listing of consumed foods; recall of forgotten foods; investigation of the time or the occasion of each meal; search for more detailed information) [28], while in NHANES 2002, the evaluation was performed using the automated 5-step multiple-pass method of the US Department of Agriculture (USDA) [28,29]. In NHANES 2001–2002, the USDA food and nutrient database was used to process a dietary intake analysis. We evaluated the intake of total energy (kcal/day), carbohydrate (g/day), protein (g/day and g/kg), lipids (g/day), saturated fat (g/day), monounsaturated fat (g/day), polyunsaturated fat (g/day), fiber (g/day), alcohol (g/day), total omega-3 (g/day), vitamin A (mcg/day), vitamin E (mg/day), retinol (mcg/day), lycopene (mcg/day), combined lutein/zeaxanthin (mcg/day), β-cryptoxanthin (mcg/day), β-carotene (mcg/day), and α-carotene (mcg/day).

### 2.6. Covariates of Interest

Since some factors can confound the association between serum antioxidants and muscle strength, some variables were considered as possible confounders in this association. In relation to demographic parameters, age (years), race/ethnicity (non-Hispanic white, non-Hispanic black, Mexican American, other Hispanic, other races), sex (men or women), marital status (single/divorced/widowed/never married or married/living as married), annual household income (0 to $19,999, from $20,000 to 54,999, or above $55,000), and educational level (under/high school graduate and some college or over), were included. Habits and health conditions were self-reported by the participants and included: hypertension (yes or no), diabetes (yes, no, or pre-diabetes), smoking (yes or no), and arthritis (yes or no). In addition, physical activity was analyzed according to intensity, divided into moderate (yes or no) and vigorous (yes or no), and strength exercise (yes or no). Regarding biochemical parameters, uric acid (mg/dL) and C-reactive protein (CRP) (mg/dL) were considered confounders. In the dietary intake, energy (kcal/day) and protein (g/day) intakes were included. BMI was considered a confounder of anthropometric data. 

### 2.7. Statistical Analysis

Sociodemographic, health conditions and habits, anthropometry, muscle strength, serum antioxidants, and biochemical and dietary intake characteristics were described for the total sample and according to sex. Mean and standard deviation were used to describe continuous variables, whereas percentage and confidence interval were used for categorical variables. In addition, a missing category was created to categorize participants who had missing data in some variables (marital status, annual family income, education, moderate and vigorous physical activity, strength physical activity, smoking, hypertension, menopause). Linear regression was used to estimate the coefficients and 95% confidence intervals (95% CI) for peak strength (muscle strength), by tertile of serum antioxidants. Analyses were performed without adjustments (Model 1) and with adjustments for confounders (co-variates of interest). A statistical analysis was performed using the Stata 14.0 software (StataCorp, College Station, TX, USA) and *p* < 0.05 was considered statistically significant.

## 3. Results

### 3.1. Individual’s Characteristics 

The total sample characteristics and results separated by sex are shown in Table 1. Assessing the total sample, the average age was 61.4 ± 9.3 years, the individuals were predominantly men, non-Hispanic white, married/living as married, had an annual household income above US $20,000, and a high education level. Most performed moderate exercise and did not have a diagnosis of diabetes, hypertension or arthritis. In general, men and women presented similar characteristics to those reported for the total sample. 

Compared with men, women were older, had lower weight, height, and peak strength; however, they presented a higher prevalence of hypertension and arthritis. Regarding the blood parameters evaluated, women showed higher levels of vitamin E (µmol/L), α-carotene (µmol/L), trans-β-carotene (µmol/L), cis-β-carotene (µmol/L), β-cryptoxanthin (µmol/L), carotenoids (µmol/L), and C-reactive protein (mg/dl), while men had higher values of retinol (µg/dL) and uric acid (mg/dL). Regarding dietary data, women had a lower consumption of energy (kcal) and nutrients.

### 3.2. Peak Force and Tertiles of Plasma Antioxidants

Table 2 shows the linear regression between tertiles of serum antioxidants and muscle strength. In the unadjusted analyses, muscle strength was negatively associated with α-carotene, trans-β-carotene, cis-β-carotene, β-cryptoxanthin and vitamin E; meanwhile, trans lycopene was positively associated with muscle strength (Model 1). However, after adjustments for confounders, only α-carotene was positively associated with muscle strength (Model 2).

## 4. Discussions

The main result of the present study was that serum α-carotene levels, but not other antioxidants, were positively associated with muscle strength in older adults. This result suggests that the type of antioxidant seems to be important for its association with muscle strength.

The aging process leads to strength loss and one of the possible causes is the increased oxidative stress [17,18,19,20,21]. Thus, antioxidants can minimize the increase of oxidative stress in older adults and can be positively associated with muscle strength. The explanation as to why only serum α-carotene (but not all the other antioxidants) was associated with strength in the present study is not fully clear, but can be related to the antioxidant action promoted by α-carotene. Although α-carotene is chemically similar to β-carotene, some studies suggest that α-carotene may have a greater potential antioxidant effect [30,31]. For example, an in vivo study showed that α-carotene seems to inhibit the proliferation of the human neuroblastoma cells about 10 times more than β-carotene [30]. In addition, the consumption of foods with high α-carotene content, such as dark green and yellow–orange vegetables, was more strongly associated with a decreased risk of lung cancer than compared with the consumption of all other types of vegetables [32]. Lastly, serum α-carotene levels are inversely associated with the risk of death from several causes [33]. Collectively, α-carotene seems to have more important antioxidant properties when compared with other types of carotenoids, such as β-carotene. The main food predictors of α-carotene are carrots and other root vegetables [34,35], bananas, oranges, and tangerines [34,36], but α-carotene is also correlated with the total consumption of fruits and vegetables [35]. Therefore, it is possible to suggest that an increased consumption of fruits and vegetables (mainly the α-carotene food sources) may be a protective factor for muscle strength in older adults. However, longitudinal studies or randomized clinical trials should be performed to confirm this hypothesis.

In the present study, vitamin E, trans-β-carotene, cis-β-carotene, β-cryptoxanthin, carotenoids, and retinol were not associated with muscle strength, but this is not a consensus in the literature. Semba et al. [23] carried out a study with 669 older women aged between 70 and 79 years. An independent association was observed between the levels of carotenoids and α-tocopherol in the blood, and handgrip, knee, and hip extension strength. Sahni et al. [16] performed a prospective cohort study that associated the antioxidant intake (evaluated by Food Frequency Questionnaire, not serum levels) with handgrip strength. Total carotenoids, lycopene, and lutein + zeaxanthin were positively associated with strength [16]. In addition, Cesari et al. [37] observed a positive association between dietary β-carotene and knee extension strength in older adults from Italy. Differences in study design, populations, confounders added as adjustments, and forms to assess the consumption of antioxidants (diet vs. serum), may explain the controversial results between these studies [16,23,37] and ours. Nevertheless, collectively, we and others [16,23,37] show that antioxidant intake seems to be important for muscle strength, which reinforces that older adults should consume adequate servings of fruits and vegetables. 

Our study has limitations. First, it is not possible to establish causality, since this is cross-sectional study. Second, as this is an observational study, confounding factors can influence the results; however, we adjusted the regression analyses for several important confounders. Third, oxidative stress biomarkers were not evaluated, which could aid an understanding of the results. However, the strengths of this study are the method we used to assess muscle strength (isokinetic dynamometer), and the method of measuring antioxidants in the blood (high-performance liquid chromatography method). Finally, the results found can be extrapolated to the population of the United States, since this is a representative sample.

## 5. Conclusions

We conclude that α-carotene, but not other antioxidants, is positively associated with muscle strength in older adults. Future randomized clinical trials should evaluate the effect of the increased intake of α-carotene food sources on muscle strength in older adults.

## Figures and Tables

**Table 1:** Sociodemographic, anthropometric and body composition characteristics of the individuals in the total sample and according to sex. NHANES, 2001–2002.

Variables	Total	Men	Women	*p*-Value
Age, years	61.4 (9.3)	60.8 (9.0)	62.0 (9.6)	0.013
Non-Hispanic white, %	83.7 (77.6–88.3)	84.9 (77.3–90.3)	82.3 (77.0–86.6)	0.171
***Sex, %***				
Men	51.4 (49.4–53.3)			
Women	48.6 (46.6–50.6)			
***Marital status, %***				0.930
Single/divorced/widowed/never married	26.1 (22.2–30.4)	15.9 (11.8–21.0)	36.9 (31.6–42.4)	
Married/living as married	73.6 (69.3–77.6)	83.7 (78.5–87.8)	63.0 (57.5–68.3)	
Missing	0.3 (0.04–1.5)	0.4 (0.04–3.2)	0.1 (0.01–0.7)	
***Annual family income, %***				0.165
$0–19.999	17.8 (14.6–21.4)	14.3 (10.9–18.5)	21.4 (17.4–26.1)	
$20.000–54.999	40.1 (35.3–45.1)	40.2 (33.0–47.8)	40.0 (36.5–43.7)	
$55.000–74.999	40.0 (33.4–47.1)	44 (35.9–52.4)	35.9 (30.1–42.1)	
Missing	2.1 (1.2–3.4)	1.5 (0.8–2.7)	2.7 (1.4–4.7)	
***Educational level, %***				0.287
High school graduate or under	41.7 (37.2–46.2)	39.7 (33.2–46.5)	43.7 (38.9–48.7)	
Some college or above	58.3 (53.7–62.7)	60.3 (53.5–66.7)	56.2 (51.2–70.0)	
***Health conditions and habits, %***				
Hypertension	37.7 (33.3–42.2)	32.7 (27.8–38.1)	42.9 (36.9–49.0)	0.032
Missing	0.1 (0.02–0.43)	0.2 (0.04–0.8)		
Diabetes				0.173
Pre-diabetes	2.4 (1.2–4.7)	2.6 (1.05–6.5)	2.1 (0.9–4.6)	
Yes	9.0 (7.4–11.0)	10.7 (8.1–14.0)	7.3 (5.4–9.8)	
No	88.6 (85.5–91.1)	86.6 (81.5–90.5)	90.6 (87.8–92.8)	
Smoking				0.640
Yes	17.3 (14.6–20.4)	18.1 (14.4–22.4)	16.5 (13.4–20.2)	
No	82.5 (79.6–85.2)	81.8 (77.5–85.3)	83.4 (79.7–86.5)	
Missing	0.2 (0.03–0.45)	0.1 (0.02–0.8)	0.1 (0.01–0.7)	
Arthritis				0.002
Yes	35.7 (31.1–40.7)	30.9 (25.7–36.7)	40.8 (35.5–46.4)	
No	64.2 (59.3–68.9)	69.1 (63.3–74.3)	59.1 (53.6–64.5)	
***Physical activity %***				0.775
Moderate physical activity				
Yes	51.4 (45.8–56.9)	50.9 (44.7–57.1)	51.8 (42.9–60.6)	
No	48.6 (43.0–54.2)	49.1 (42.8–55.3)	48.1 (39.2–57.1)	
Vigorous physical activity				
Yes	28.9 (23.9–34.4)	32.1 (25.3–39.9)	25.4 (20.3–31.2)	
No	71.1 (65.5–76.1)	67.8 (60.1–74.7)	74.5 (68.6–79.6)	
Resistance physical activity				
Yes	24.1 (19.2–29.7)	23.4 (17.2–30.9)	24.8 (19.0–31.7)	
No	75.8 (70.2–80.7)	76.6 (69.1–82.7)	75.1 (68.2–80.9)	
Missing	0.1 (0.06–0.1)		0.1 (0.01–0.7)	
***Anthropometrics***				
Weight, kg	80.4 (18.1)	87.2 (16.0)	73.1 (17.4)	<0.001
Height, m	1.68 (0.10)	1.75 (0.07)	1.61 (0.06)	<0.001
Body mass index, kg/m^2^	28.2 (5.5)	28.25 (4.6)	28.1 (6.4)	0.813
***Strength***				
Peak force, N	384 (123,7)	457 (111)	307 (82.4)	<0.001
Time to peak force, s	1.03 (0.48)	0.99 (0.39)	1.06 (0.56)	0.067
Peak force velocity, degree/s	60.7 (0.62)	60.7 (0.67)	60.6 (0.57)	0.121
***Biochemical parameters***				
Vitamin E (µmol/L)	37.6 (15.7)	36.1 (15.1)	39.2 (16.2)	0.013
α-carotene (µmol/L)	0.09 (0.13)	0.08 (0.10)	0.11 (0.16)	0.008
Trans-β-carotene (µmol/L)	0.44 (0.43)	0.37 (0.36)	0.52 (0.48)	<0.001
Cis-β-carotene (µmol/L)	0.02 (0.02)	0.02 (0.02)	0.03 (0.03)	<0.001
β-cryptoxanthin (µmol/L)	0.18 (0.13)	0.16 (0.12)	0.19 (0.14)	0.017
Combined Lutein/zeaxanthin (µmol/L)	0.31 (0.17)	0.30 (0.16)	0.32 (0.18)	0.163
Trans-lycopene (µmol/L)	0.41 (0.21)	0.42 (0.22)	0.41 (0.20)	0.834
Carotenoids (µmol/L)	1.46 (0.79)	1.35 (0.70)	1.58 (0.87)	0.002
Retinol (µg/dL)	67.1 (1.02)	69.2 (1.04)	64.9 (1.20)	<0.001
C-reactive protein (mg/dL)	0.41 (0.03)	0.35 (0.03)	0.48 (0.04)	<0.001
Uric acid (mg/dL)	5.58 (1.41)	6.10 (1.29)	5.03 (1.31)	<0.001
***Dietary intake***				
Energy, kcal	1997 (960)	2313 (1112)	1662 (609)	<0.001
Carbohydrate, g	245 (126)	278 (149)	210 (82.1)	<0.001
Protein, g	75.7 (39.4)	87.0 (44.8)	63.7 (28.1)	<0.001
Protein, g/kg	0.97 (0.49)	1.01 (0.51)	0.92 (0.47)	0.006
Lipids, g	77.5 (47,9)	90.4 (56.6)	63.9 (31.6)	<0.001
Saturated fat, g	24.1 (16.2)	28.4 (19.1)	19.5 (10.8)	<0.001
Monounsaturated fat, g	27.7 (18.7)	32.7 (22.1)	22.5 (12.3)	<0.001
Polyunsaturated fat, g	16.4 (12.1)	18.5 (14.2)	14.2 (8.9)	<0.001
Total omega-3, g	1.80 (1.50)	2.04 (1.71)	1.55 (1.18)	0.001
ALA, g	1.50 (1.21)	1.68 (1.33)	1.32 (1.03)	0.001
EPA, g	0.05 (0.19)	0.06 (0.21)	0.04 (0.16)	0.198
DHA, g	0.09 (0.30)	0.11 (0.36)	0.07 (0.22)	0.119
Fiber, g	16.2 (10.4)	17.5 (12.0)	14.8 (8.1)	0.010
Alcohol, g	8.68 (24.1)	12.4 (29.0)	4.72 (16.8)	<0.001
Vitamin A, RAE (mcg)	629 (719)	670 (886)	586 (480)	0.086
Vitamin E, as α-tocopherol (mg)	7.07 (5.75)	7.85 (6.64)	6.25 (4.51)	0.002
Retinol (mcg)	416 (586)	467 (757)	361 (307)	0.002
Lycopene (mcg)	5520 (10271)	6108 (11183)	4899 (9179)	0.029
Lutein + zeaxanthin (mcg)	1717 (3118)	1760 (3285)	1672 (2934)	0.705
β-cryptoxanthin (mcg)	146 (229)	157 (253)	134 (201)	0.128
β-carotene (mcg)	2271 (4185)	2125 (4391)	2425 (3954)	0.322
α-carotene (mcg)	412 (1480)	428 (1766)	395 (1100)	0.738

Data are described as mean (standard deviation) or percentage (confidence interval). Notes: DHA, docosahexaenoic acid; EPA, eicosapentaenoic acid, ALA: alpha linolenic acid, RAE, retinol activity equivalents.

**Table 2:** Linear regression between tertiles of serum antioxidants and muscle strength. NHANES, 2001–2002.

	Model 1 β (95% CI)				Model 2 β (95% CI)			
	T1	T2	T3	*p*-Trend	T1	T2	T3	*p*-Trend
α-carotene (µmol/L)	Ref	2.37 (−17.7; 22.5)	−18.0 (−32.1; −3.90)	**0.016**	Ref	15.1 (2.24; 28.0)	16.0 (1.89; 30.1)	**0.027**
Trans-β-carotene (µmol/L)	Ref	1.24 (−15.5; 18.0)	−42.6 (−64.8; −20.4)	**0.001**	Ref	11.1 (−2.83; 25.1)	9.13 (−9.41; 27.7)	0.316
Cis-β-carotene (µmol/L)	Ref	−17.6 (−30.8; −4.42)	−50.2 (−68.7; −31.6)	**<0.001**	Ref	4.70 (−7.61; 17.0)	7.33 (−11.0; 25.7)	0.399
β-cryptoxanthin (µmol/L)	Ref	−17.1 (−39.6; 5.41)	−23.9 (−47.9; 0.05)	**0.042**	Ref	6.73 (−10.2; 23.7)	−0.289 (−13.0; 12.4)	0.990
Combined Lutein/zeaxanthin (µmol/L)	Ref	19.0 (−1.78; 39.7)	1.25 (−24.1; 26.6)	0.872	Ref	11.8 (−6.15; 29.7)	6.95 (−14.0; 27.9)	0.473
Trans-lycopene (µmol/L)	Ref	21.9 (4.25; 39.6)	28.7 (9.0; 48.5)	**0.010**	Ref	11.8 (−5.24; 28.9)	3.32 (−9.20; 15.8)	0.740
Carotenoids (µmol/L)	Ref	9.90 (−12.5; 32.3)	−14.1 (−37.7; 9.42)	0.222	Ref	0.10 (−17.8; 18.0)	0.17 (−18.3; 18.6)	0.985
Vitamin E (µmol/L)	Ref	0.52 (−19.2; 20.2)	−22.9 (−41.9; −3.87)	**0.019**	Ref	2.26 (−8.89; 13.4)	8.71 (−8.00; 25.4)	0.282
Retinol (µg/dL)	Ref	−2.36 (−29.4; 24.7)	21.4 (−10.1; 52.9)	0.159	Ref	8.21 (−5.62; 22.0)	11.1 (−12.8; 35.0)	0.358

Model 1: unadjusted analyses. Model 2: adjusted for energy (kcal/day) and protein (g/day) intake, body mass index, sex, age, C-reactive protein (mg/dL), uric acid (mg/dL), race/ethnicity, marital status, annual household income, educational level, physical activity, smoking, hypertension, arthritis and diabetes. Bold means that the *p*-value is statistically significant.

## Data Availability

Data can be found in “https://wwwn.cdc.gov/nchs/nhanes/continuousnhanes/default.aspx?BeginYear=2001”.
